# Association between SNP rs527616 in lncRNA *AQP4-AS1* and susceptibility to breast cancer in a southern Brazilian population

**DOI:** 10.1590/1678-4685-GMB-2020-0216

**Published:** 2021-03-12

**Authors:** Rafael D. Marchi, Carolina Mathias, Gabriel A. K. Reiter, Rubens Silveira de Lima, Flávia Kuroda, Cícero de Andrade Urban, Ricardo. L. R. de Souza, Daniela F. Gradia, Enilze M. S. F. Ribeiro, Iglenir J. Cavalli, Jaqueline Carvalho de Oliveira

**Affiliations:** 1 Universidade Federal do Paraná, Departamento de Genética, Curitiba, PR, Brazil.; 2 Hospital Nossa Senhora das Graças, Centro de Doenças da Mama, Curitiba, PR, Brazil.

**Keywords:** rs527616, lncRNA *AQP4-AS1*, breast cancer, case-control study, Brazilian population

## Abstract

Breast cancer (BC) is the leading cause of death by this disease in women worldwide. Among the factors involved in tumorigenesis, long non-coding RNAs (lncRNAs) and their differential expression have been associated. Differences in gene expression may be triggered by variations in DNA sequence, including single nucleotide polymorphisms (SNPs). In the present study, we analyzed the rs527616 (C>G), located in the lncRNA *AQP4-AS1*, using PCR-SSP in 306 BC patients and 312 controls, from a Brazilian population. In the BC group, the frequency found for CG heterozygotes was above the expected and the overdominant model is the best one to explain our results (OR: 1.70, IC 95%: 1.23-2.34, P<0.001). Furthermore, the SNP were associated with age at BC diagnosis and the risk genotype more frequent in the older age group. According to TCGA data, *AQP4-AS1* is down-regulated in BC tissue, and the overexpression is associated with better prognoses, including Luminal A, HER2-, stage 1 of disease and smaller tumor. In conclusion, the CG genotype is associated with increased susceptibility in the southern Brazilian population. This SNP is mapped in the lncRNA *AQP4-AS1*, showing differential expression in BC samples. Based on these results, we emphasize the potential of the role of AQP4-AS1 in cancer.

## Introduction

Breast cancer is the most commonly diagnosed neoplasm in women worldwide (Bray *et al.*, 2018). In Brazil, it is the second most recurrent type of cancer in women after non-melanoma skin cancer (INCA, 2020). Despite the improvement in prevention, diagnosis, and classification methods, there is still a high mortality rate (Bray *et al.*, 2018), which justifies the search for new prognostic markers, among which analysis of non-coding RNAs stand out.

LncRNAs are non-coding RNAs with more than 200 nucleotides in length, with essential regulatory roles in several biological processes and associated with many pathological conditions (Cipolla *et al.*, 2018). There are more than 17,000 lncRNA genes described in the human genome (Frankish *et al.*, 2019). Despite the large number of lncRNAs identified, many of them have unknown functions. Additionally, genomic variants, including single nucleotide polymorphisms (SNPs), may contribute to modifying the functioning of lncRNAs, thus affecting cancer susceptibility (Wapinski and Chang, 2011) but there are few studies focused on these regions, showing that this is still an underexplored field. 

Located in the region of *AQP4-AS1*, the SNP rs527616 (C> G), has been indicated by genome-wide association studies (GWAS) (Michailidou *et al.*, 2017) as being associated with an increased risk of developing breast cancer, but this variation has not been deeply investigated. 

The *AQP4-AS1* gene (Aquaporin 4 antisense RNA 1) transcribes an antisense lncRNA of unknown function (Halladay *et al.*, 2018). As many antisense transcripts may regulate the host transcript (Wight and Werner, 2013), the nearby aquaporin 4 gene (*AQP4*) may help us to understand the role of this lncRNA. 

AQP4 has a fundamental role in maintaining water homeostasis, which is believed to be associated with the development of tumors (Li et al., 2016). In breast cancer, AQP4 is low expressed in comparison to non-tumor tissues and associated with prognosis (Shi *et al.*, 2011; Zhu *et al.*, 2019). 

By knowing the importance of AQP4 in breast cancer, we aimed to perform a case-control study to evaluate the association of the SNP rs527616 (C> G) with breast cancer susceptibility in a southern Brazilian population, and to further evaluate the *AQP4-AS1* expression in public data. 

## Subjects and Methods

### Study cohort

The analyses were performed using tumor samples of 306 patients with sporadic breast cancer from the *Hospital Nossa Senhora das Graças* (HNSG), located in Curitiba, in the South of Brazil. As control group, we used peripheral blood samples of 312 women with no cancer history, from the biobank of the Department of Genetics at Federal University of Paraná (UFPR), Curitiba, Brazil. 

Both groups (patients and controls) were from the same region in the south of Brazil, most living in the metropolitan region of Curitiba, Parana State. Ancestry information was obtained from self-reported patients’ records, with 84.7% white, 10.7% black or brown, and 1.9% others. 

Although genomic information to assess ancestry was not available for all individuals, previous studies showed that, in this Brazilian region and in accordance with phenotypic classification, the white population is of predominantly of European ancestry (more than 80% contribution) and the black/brown population consists predominantly of African (~50%) and European (~42%) ancestry, with a smaller contribution of Amerindian (~8%) ancestry (Probst *et al.*, 2000, Braun-Prado *et al.*, 2000).

A subset of patients, also included in the present study, was genotyped using a SNP chip Illumina Infinium QC Array (Illumina Inc., CA), which contains 15,949 markers (including ~3,000 ancestral informative markers (AIMs) and, based on the results previously shown, the genetic analysis was able to differentiate the two main population groups, European (EUR) and African (AFR) in our samples, thus confirming the self-report ethnicity information (Sugita *et al.*, 2016).

The mean ages of the case and the control groups were 56.23 ± 15 and 47.66 ± 4.69. Histopathological parameters are summarized in Table 1. The immunohistochemical classification was based on Goldhirsch *et al.* (2013). The samples were collected under the approval of the Human Research Ethics Committee of the Health Sciences Sector of UFPR, under the number CAAE: 67029617.4.0000.0102. All participants signed an informed written consent.

**Table 1 -:** Clinical and Histopathological Data of Breast Cancer patients.

Breast cancer cases n = 306					
Histology	n	%	Tumor Grade	n	%
Ductal	209	68%	I	22	7%
Lobular	30	10%	II	115	38%
Mucinous	8	3%	III	59	19%
Mixed duct-lobular	17	6%	Without information	110	36%
Others	29	9%			
Without information	13	4%			
Immunohistochemical Subtype	n	%	Lymph node metastasis	n	%
Luminal A	79	26%	Presence	86	28%
Luminal B	132	43%	Absence	176	58%
HER2 positive	17	6%	Without information	44	14%
Triple-negative	29	9%			
Without information	49	16%			

### Genotyping

DNA extraction was performed by the phenol-chloroform method in tissue samples. The peripheral blood DNA from women with no cancer was extracted by the salting-out method and used as control (Serino *et al.*, 2019)

**Figure 1 -:**
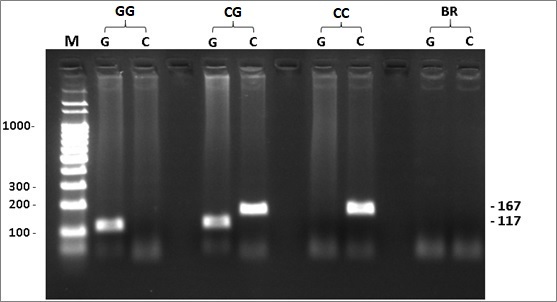
Electrophoretic pattern of allele specific PCR of rs527616 (C>G), located in the lncRNA *AQP4-AS1.* M: Molecular weight marker, GG: Homozygous sample, CG: heterozygous sample, CC: homozygous sample, BR: white control. Expected fragment size: C-167 bp and G-117 bp.

The SNP rs527616 genotyping was performed by PCR with specific sequence primers (PCR-SSP), using a set of specific primers for the recognition of each allele. Allele C: Forward 5’GCTCCAGTGCTATTTG3 ‘and Reverse 5’ACAGGTCAAGGAAATGC3’, yielding a product with the size of 167 bp. Allele G: Forward 5 ‘GTTGTAGAAGGCACAGTTG3’ and Reverse 5 ‘AGGACAAGTCTAAACTAGGG3’, yielding a product with the size of 117 bp. PCRs were performed from 2 μl of DNA in a concentration of 20 ng/μl and 160 pmol of specific primer in the presence of Master Mix for conventional PCR (1x), containing 0.2 mM dNTPs, 50 mM KCl, 10 mM Tris-HCl and 1.25 U *Taq* polymerase, developed by IBMP, ICC / FioCruz. The PCR conditions were: 95 °C for 10 min, followed by 35 cycles of 96 °C for 30 s, 60 °C for 30 s, 72 °C for 30 s and ending with a cycle at 70 °C for 10 min. For each PCR performed, a heterozygous sample with the confirmed genotype and a negative control were included with the aim to ensure that there were no contamination and genotyping errors. The results were interpreted after electrophoresis analysis on 2% agarose gel stained with Gel Red Biotium (Figure 1). 

PCR-SSP method had high sensitivity and all individuals were genotyped. We validated the specificity and accuracy of our PCR-SSP method by sequencing samples containing the genotype homozygotes CC / GG and the heterozygotes CG with Sanger method. 

### Statistical analysis

By using the allele frequencies published by GWAS, we performed the sample size calculation, considering the 95% confidence interval and the prevalence of less frequent alleles in 20% of the population. We estimated the minimum sample size of 300 patients and 300 controls required for reliable production data (Beiguelman, 1988). For the genotypic frequency tests of the control and patient groups, we used the test of deviations in the proportions of the Hardy-Weinberg theorem by Chi-square. Additionally, we used the odds ratio (OR) calculation, as well as the Chi-Square test to assess whether the variables (breast cancer and SNPs) are independent.

Considering the overdominant model, we used FunModeling package to find the point (cut-off) with the most significant split according to age at diagnosis (44 years-old) and calculated the OR in both groups. Logistic regression was also used to confirm the role of the SNP in the overdominant model and age association.

Statistical analyses were performed with R software with the Nortest and readxl packages (Gross and Ligges, 2015; Wickham *et al.*, 2019). For all tests described above, P-values <0.05 were considered significant.

### Expression analysis in public data

Expression analysis of *AQP4-AS1* in breast cancer was performed using the RNA-Seq data available from The Cancer Genome Atlas Program (TCGA) (Cancer Genome Atlas Network). RNA-seq dataset, after normalization and log-transformation, were assessed by open-access web resource The Atlas of Noncoding RNAs in Cancer (TANRIC, https://ibl.mdanderson.org/tanric/_design/basic/main.html).

We analyzed *AQP4-AS1* expression level of 837 BC patients, and 105 non-tumor tissue through Limma R package (Smyth *et al.*, 2002) and GraphPad Prism8 using parametric *t* test. We also compared the expression level of *AQP4-AS1* according to the BC molecular classification, presence of receptors, disease stage, and tumor size. This analysis comprises 388 luminal A, 177 luminal B, 66 HER2-enriched, and 127 basal-like using ANOVA parametric test followed by Tukey test or *t* test. 

## Results

### The presence of the CG genotype in rs527616 is associated with breast cancer risk

From our genotyping results, we verified that the C allele is the least frequent one in both of the groups analyzed with minor allele frequency (MAF) of 0.30 in the patients’ group and 0.29 in control group, with no statistical difference (P = 0.92). On the other hand, the genotype heterozygote CG is more frequent in the patients group, and the homozygotes CC and GG are more frequent in the control group. 

Additionally, we calculated the OR for the recessive, dominant, and overdominant models (Table 2). The homozygotes are associated with lower risk and heterozygote, with a higher risk of BC.

**Table 2 -:** Genotype and allele frequencies of rs527616 in patients and controls.

	Patients (n=306)	controls (n=312)		
	n (%)	n (%)	*p*	OR 95%CI
CC	9 (3%)	25(8%)	0.004	0.34 (0.15-0.75)
CG	167 (55%)	129 (41%)	0.0009	1.7 (1.23-2.34)
GG	130 (42%)	158 (51%)	0.035	0.71 (0.52-0.98)
Models				
Dominant				
GG	130 (42%)	158(51%)		
CG/CC	176 (58%)	154(49%)	0.04	1.38 (1.01-1.90)
>Recessive				
GG/CG	297 (97%)	287 (92%)		
CC	9 (3%)	25 (8%)	0.004	0.34 (0.15-0.75)
>Overdominant				
CG	167 (55%)	129 (41%)		
GG/CC	139 (45%)	183 (59%)	0.0009	1.70 (1.23-2.34)
MAF (C)	185 (30%)	179 (29%)	0,57	1,07 (0.84-1.37)

MAF = minor allele frequency; *p* = P-value; OR = odds ratio; 95% CI = 95% confidence interval. Control group has no deviation in the proportions of the Hardy-Weinberg equilibrium.

### Rs527616 is associated with age at diagnosis

The SNP was significantly associated with age at the BC diagnosis. The risk genotype, CG, is more frequent in older age group. The age stratification (age ≤ 44 years and > 44 years) showed that the risk effect of the [CG] genotype of rs527616 was mainly in the older age group (> 44 years of age) with slightly more increased risk ([CG] *vs*. [CC, GG]: OR = 1.89 (1.33-2.67); P = 0.0002, Table 3). In contrast, in the younger age group (≥44 years of age), the genotype frequencies showed no significant association with BC.

**Table 3 -:** Distribution of patients with genotypes CG and GG + CC in overdominant model based on age of diagnosis.

	≤ 44 years (n=58)	Controls (n=312)		
	n (%)	n (%)	*p*	OR 95%CI
CC	2 (3.5 %)	25 (8.0 %)		
CG	25 (43.1 %)	129 (41.4 %)		
GG	31 (53.4 %)	158 (50.6 %)		
Overdominant				
CG	25 (43.1 %)	129 (41.4 %)		
GG/CC	33 (56.9 %)	183 (58.6 %)	1.07	0.93 (0.60-1.89)
Patients with ≤ 44 years-old at diagnosis				
	> 44 years (n=224)	Controls (n=312)		
	n (%)	n (%)	*p*	OR 95%CI
CC	5 (2.2 %)	25 (8.0 %)		
CG	128 (57.2 %)	129 (41.4 %)		
GG	91 (40.6 %)	158 (50.6 %)		
Overdominant				
CG	128 (57.2 %)	129 (41.4 %)		
GG/CC	96 (42.8 %)	183 (58.6 %)	0.0002	1.89 (1.33-2.67)

Patients with more than 44 years-old at diagnosis.

The allele and the genotype frequency are not associated with clinical variables in the present study. We analyzed association with subtypes, including luminal and triple negative (P = 0.14), histopathological parameters: invasion of regional lymph nodes (P = 0.16), and degree of tumor differentiation (P = 0.65).

### *In silico* gene expression analysis 

**Figure 2 -:**
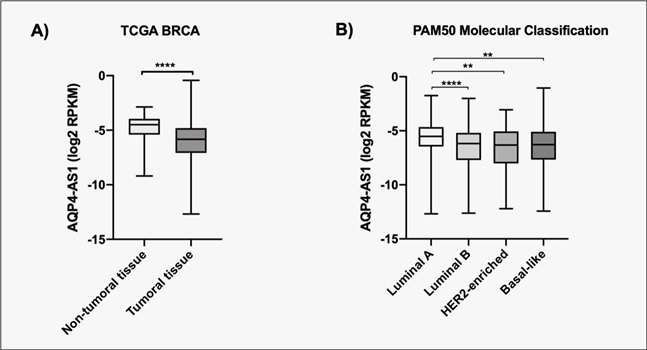
Expression of AQP4-AS1 in TCGA data. **A**. Expression of *AQP4-AS1* in non-tumoral tissue and tumor. **B.** Expression of *AQP4-AS1* in different molecular subtypes of breast cancer. ** p < 0.001 *** p < 0.0004, **** p < 0.0001.

**Figure 3:**
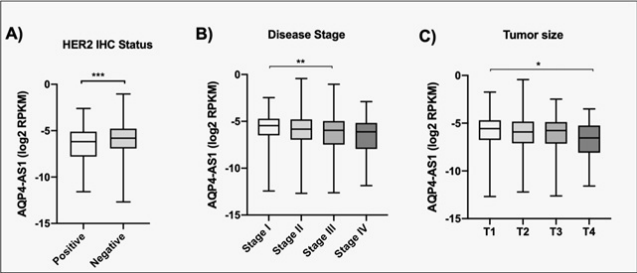
Expression of *AQP4-AS1* according to immunohistochemical (IHC) markers, stage and size of tumor. **A.** Epidermal growth factor receptor 2 (HER2) status, **B.** Stage of disease and **C.** Size of Tumor, dates from TCGA RNA-seq data*.* * p < 0.05, ** p < 0.001 *** p < 0.0004.

According to TCGA expression data, *AQP4-AS1* is down-regulated in BC tissue compared to the non-tumoral counterpart (Figure 2A), and the molecular subtype luminal A has a high level of the lncRNA in comparison with the other subtypes (Figure 2B). Besides the molecular classification, we examined the expression of *AQP4-AS1*, taking into consideration the mainly used immunohistochemical markers, tumor size, and disease stage (Figure 3).

*AQP4-AS1* is also highly expressed in groups of usual better prognosis, including HER2 negative, stage 1 of disease, and smaller tumor size (T1). These results suggest that the low expression of *AQP4-AS1* may be a common event in BC, and the high expression is associated with a better prognosis. 

## Discussion

Growing evidence suggests that SNPs may have paramount importance in genetic susceptibility to breast cancer (Li *et al.*, 2019), but SNPs in lncRNA *loci* are underexplored.

Michailidou and colleagues, in a GWAS, presented an association between breast cancer and the SNP rs527616 in European and East Asian ancestry population (Michailidou *et al.*, 2013; Michailidou *et al.*, 2017). In the present study, we searched for this association in a cohort from the South of Brazil, in a case-control study. 

The minor allele frequencies (MAF) in the Brazilian control group is C = 0.3, similar to the global population frequency MAF=0.34 (Phan *et al.*, 2020). The data released by GWAS showed an association between the risk of breast cancer and the allele (G) (OR= 1.03, CI 1.02-1.05, P <0.001) (Michailidou *et al.*, 2017). 

The GWAS usually includes a massive number of samples and *loci*, but it does not deepen the evaluation of a specific *locus*. For example, in the rs527616 analysis, only allele frequency was compared, while the influence of genotypes on BC susceptibly was not assessed. On the other hand, herein, we emphasized the heterozygote genotype in BC risk association. 

In the BC group, we observed a frequency above the expected for CG heterozygotes and below the expected for CC and GG homozygotes; but no allele association was found in our Brazilian cohort. Analyzing only allele frequency, Zhang *et al.* (2014), also did not find any BC association in Chinese women. 

Our data suggest that CC is a protective genotype and that the heterozygote CG is associated with increased susceptibility to breast cancer, thus reinforcing the importance of evaluating the influence of genotypes. As to the genotype GG, although it is significant, the 95% confidence interval range is close to 1, so it must be interpreted with caution. 

APQ4-AS1 lncRNA was not previously studied, and description about secondary structure, sites of interaction with other molecules and mechanisms of action are absent. Therefore, it is difficult to hypothesize the selective mechanism for the heterozygous genotype. However, bearing in mind that SNPs can change the structure of a lncRNA - and a secondary structure is essential for its role - in heterozygotes, both molecules are expressed simultaneously and this could amplify the possible interactions and also act differently in cell context. But further studies are essential for a better characterization of mechanism of action of this lncRNA.

A limitation of the present study is the absence of genomic information to assess ancestry for all individuals. We approached this issue including the self-reported patient records on ancestry. Considering the population analyzed, previous studies characterized the genetic background and, in accordance with self-phenotypic classification, this population is predominantly made up of European origin individuals (more than 80% of contribution).

The allele and genotype frequencies are not associated with clinical variables in the present study. This SNP was not associated with disease-free survival of triple-negative BC patients (Yuan *et al.*, 2017), or with estrogen, HER-2 status, and BC subtypes (Zhang *et al.*, 2014).

On the other hand, rs527616 was also associated with age, showing higher frequency of the CG risk genotype among older BC diagnosed patients. The heterogeneity of BC by age is well known, most notably for the high frequency of germinative mutations in younger patients and for the rising rates of hormone responsive subtypes and important lifestyle/reproductive factors in older patients (Diab *et al.*, 2000; Momenimovahed and Salehiniya, 2019). The risk genotype could be associated with a mechanism more involved in this group of patients, but further details in lncRNAs mechanism of action are important to help improve knowledge about this relation. 

Older BC diagnosed patients are usually associated with better prognosis and, in expression analysis, higher expression of *AQP4-AS1* in patients were also associated with better outcome groups. 

According to the expression data, there is a reduction in the expression of *AQP4-AS1* in the tumor tissue in comparison with the non-tumor tissue and the higher expression in luminal A subtype in comparison with the other subtypes. In addition, its expression was higher in patients in the first stage and minor tumor size, suggesting its relation with a better prognosis. *AQP4-AS1* expression was not previously analyzed in breast cancer, but the gene *AQP4* expression has the same profile of the *AQP4-AS1*, with low expression in tumor and the expression associated with better prognosis (Shi *et al.*, 2011; Zhu *et al.*, 2019). 

Aquaporins (AQPs) are a family of small membrane transport proteins that act as selective pores for water and small solutes (Verkman *et al.*, 2008; Mobasheri and Barrett-Jolley, 2013). More specifically, AQP4 has a fundamental role in maintaining water homeostasis and it can be associated with the development of cancer (Li *et al.*, 2016). 

In breast cancer, AQP4 had a low expression in comparison with non-tumor tissues, and the patients with the lowest expression level had poor survival (Shi *et al.*, 2011; Zhu *et al.*, 2019). Additionally, down-regulation of AQP4 inhibits proliferation, migration, and invasion in breast cancer cell lines (Li et al., 2016). 

As many antisense lncRNAs act regulating the host gene, this may be a mechanism for the role of *AQP4-AS1*. It is known that antisense genes can alter the expression of sense genes in several ways, such as DNA methylation, chromatin modification, variation of isoforms, and alteration of RNA stability (Pelechano and Steinmetz, 2013). However, further studies need to be carried out to elucidate the interactions of this lncRNA.

Additionally, the homozygote genotypes are less frequent in tumor samples, thus it would be interesting to check if SNPs genotypes are associated with different expression levels. The above suggestion is feasible since it is known that SNPs can interfere in the expression of a gene by changing the structure of a lncRNA, also on its binding site to proteins and secondary mechanisms of the corresponding messenger RNAs, or even by changing its interaction (Li *et al.*, 2019).

Our results are relevant to emphasize the potential of the role of AQP4-AS1 lncRNAs role in breast cancer. In conclusion, we describe for the first time in a Brazilian population that the rs527616 polymorphism (C>G) is associated with breast cancer susceptibility, with CG as the risk genotype and CC as the genotype with protective effect. Furthermore, AQP4-AS1 has low expression in BC samples and high expression groups of better prognoses: luminal A, HER2 negative, stage 1, and tumor size T1.
